# Review on the Mechanism of and Therapies Targeting PANoptosis in Ulcerative Colitis

**DOI:** 10.3390/biom16050624

**Published:** 2026-04-22

**Authors:** Mi Zhao, Min Liu, Wen Tian, Tiantian Ren, Jianing Jing, Ya Zheng, Zhaofeng Chen

**Affiliations:** 1 The First Clinical Medical College, Lanzhou University, Lanzhou 730000, China; zhmi2024@lzu.edu.cn (M.Z.);; 2 Department of Gastroenterology, The First Hospital of Lanzhou University, Lanzhou 730000, China; 3 Gansu Province Clinical Research Center for Digestive Diseases, The First Hospital of Lanzhou University, Lanzhou 730000, China

**Keywords:** ulcerative colitis (UC), PANoptosis, targeted therapy, multi-target inhibition, smart hydrogel

## Abstract

Ulcerative colitis (UC) is a complex chronic inflammatory bowel disease, and its pathogenesis is closely related to immune imbalance, intestinal flora disorder and intestinal barrier damage. In recent years, a novel form of programmed cell death, PANoptosis, has been confirmed to play a core role in the pathological process of UC. PANoptosis is driven by the PANoptosome complex, which is assembled by key molecules such as ZBP1, NLRP3, and RIPK1, which can simultaneously activate pyroptosis, apoptosis, and necroptosis. This not only leads to damage to the intestinal epithelial barrier, but it also aggravates the dysfunction of immune cells by releasing a large amount of pro-inflammatory cytokines and damage-associated molecular patterns (DAMPs), thus forming a vicious cycle of “cell death and inflammation”. Given the complexity of the PANoptosis signaling network, the efficacy of single-target inhibitors is limited. This review systematically expounds the mechanism of action of PANoptosis in UC and focuses on discussing multi-target combination treatment strategies represented by smart hydrogels loaded with multiple inhibitors (such as MCC950, GSK772, VX-765, disulfiram, etc.). This strategy achieves synergy through “vertical blocking” and “horizontal coverage”, and in combination with targeted delivery to the lesion, provides a highly promising innovative direction for fundamentally breaking the pathological cycle of UC. Future research should focus on the development of new inhibitors, the optimization of delivery systems, and in-depth clinical translation to promote this strategy as a breakthrough therapy for refractory UC.

## 1. Introduction

UC is a chronic and recurrent inflammatory bowel disease with a complex etiology. The continuous increase in its global incidence has made it a major public health challenge. The typical pathological features of UC include persistent damage to the intestinal mucosal barrier, significant imbalance between the innate and adaptive immune systems, and dysbiosis of the intestinal microbiota [1]. Although current treatment strategies, such as aminosalicylate preparations, glucocorticoids and biologics, can alleviate inflammation to a certain extent, a large number of patients still face many difficulties such as insufficient efficacy, drug resistance or adverse drug reactions [2]. This clinical situation reveals that there are key links in the pathogenesis of UC that have not been fully clarified. We need new theoretical breakthroughs to bring about more effective treatment methods.

In recent years, the role of programmed cell death in driving chronic inflammatory diseases has received increasing attention. Among them, PANoptosis, a newly discovered form of inflammatory cell death that integrates the core features of pyroptosis, apoptosis and necroptosis, provides a brand new perspective for understanding the pathological process of UC. Its molecular basis lies in the fact that PANoptosome is a dynamically assembled multi-protein complex, which can integrate signals from various sensors such as NLRP3, ZBP1 and RIPK1, and then cooperatively activate caspase family and RIPKs, ultimately leading to irreversible damage to the cell membrane (through pore-forming proteins such as GSDMD and MLKL) and the release of a large amount of pro-inflammatory cytokines and DAMPs [3]. This powerful cell death pattern not only directly leads to the disintegration of the intestinal epithelial barrier, but also triggers and amplifies uncontrolled immune inflammation, forming a vicious cycle of “cell death, inflammation, more cell death”. This might be one of the core mechanisms underlying the chronicity and recurrent attacks of UC [3,4].

Therefore, targeting the PANoptosis signaling pathway has become a highly promising direction in the treatment of UC. However, due to the complexity and interactivity of the PANoptosis signaling network, inhibitors targeting a single molecule (such as NLRP3, RIPK1, or caspase-1) often show limited efficacy in clinical trials. In view of this, the development of multi-target combination inhibition strategies that can simultaneously intervene at multiple key nodes of PANoptosis, and the use of intelligent drug delivery systems (such as colon-targeted responsive hydrogels) to achieve precise and synergistic drug delivery at the lesion site have demonstrated great application prospects [5]. This review aims to systematically expound the core mechanism underlying PANoptosis’ role in the occurrence and development of UC, and focuses on discussing innovative treatment strategies represented by multi-target inhibitors loaded in hydrogels, providing new theoretical bases and research and development ideas for conquering this refractory disease. Although direct detection of the assembled PANoptosome complex in human UC biopsies remains technically challenging due to its transient and dynamic nature, accumulating evidence has demonstrated elevated expression of its core components—such as NLRP3, GSDMD, and RIPK3—in the colonic mucosa of UC patients, supporting the clinical relevance of this pathway [6].

## 2. The Mechanism of UC

**Figure 1:**
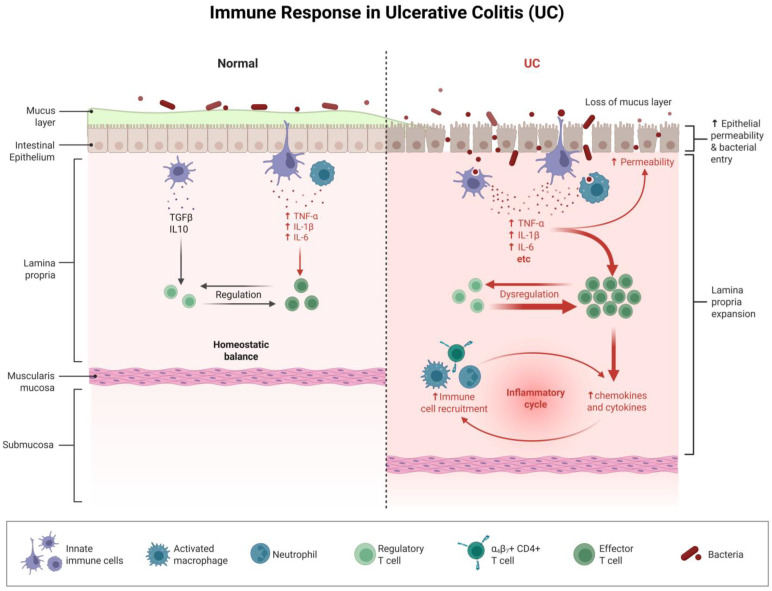
In the normal intestine, the mucus layer and intestinal epithelium are intact. Within the lamina propria, regulatory T cells, innate immune cells, etc., maintain homeostatic balance through factors such as TGFβ and IL10. In UC, the mucus layer is lost, and the intestinal epithelial permeability increases, allowing bacteria to invade and triggering innate immune cells and activated macrophages to release a large number of pro-inflammatory factors (such as TNF-α, IL-1β, IL-6, etc.). This leads to the dysfunction of regulatory T cells and the massive expansion of effector T cells. At the same time, α_4_β_7_^+^ CD4^+^ T cells and others promote immune cell recruitment, with neutrophils participating in the process, forming an inflammatory cycle. This results in further release of chemokines and cytokines, exacerbating lamina propria expansion and intestinal inflammation, reflecting the series of pathological changes caused by immune response dysregulation in UC.

UC is a chronic non-specific intestinal inflammatory disease with an unclear etiology. It mainly affects the rectal and colonic mucosa, with persistent or recurrent diarrhea, mucus and bloody stools, and abdominal pain as the main clinical features. Its pathogenesis is complex, involving multiple key links such as immune imbalance, intestinal flora disorder, and intestinal barrier damage. These links interact with each other, forming a vicious cycle [7]. In terms of immune imbalance, the abnormal activation of innate and adaptive immune cells is the core driving force for the onset of UC. Innate immune cells such as neutrophils and macrophages, upon stimulation by the intestinal microbiota or damage signals, will extensively infiltrate the intestinal mucosal tissue and release a variety of pro-inflammatory cytokines such as tumor necrosis factor-α (TNF-α), interleukin-1β (IL-1β), and interleukin-6 (IL-6), initiating an inflammatory response [8]. Th1 cells are adaptive immune cells that can secrete interferon-γ (IFN-γ), activate immune cells such as macrophages, and further promote the release of inflammatory factors. Th17 cells can secrete interleukin-17 (IL-17), interleukin-22 (IL-22), etc., recruit neutrophils to infiltrate, and damage the intestinal epithelial barrier. The abnormal differentiation and hyperfunction of these adaptive immune cells jointly drive the chronic inflammatory process of UC. Meanwhile, functional defects in regulatory T cells (Treg) lead to a reduction in the secretion of anti-inflammatory factors such as interleukin-10 (IL-10) and transforming growth factor-β (TGF-β), which therefore cannot effectively suppress the excessive immune responses, thereby aggravating the immune imbalance [9] (Figure 1). 

**Figure 2:**
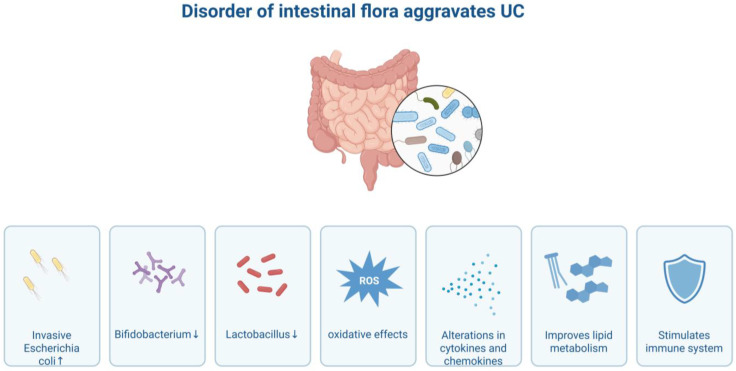
This figure illustrates how disorder of the intestinal flora aggravates ulcerative colitis (UC). It shows the human intestine with a magnification of intestinal flora, and lists several key changes: an increase in invasive *Escherichia coli*, a decrease in beneficial bacteria like *Bifidobacterium* and *Lactobacillus*, the occurrence of oxidative effects (indicated by ROS), alterations in cytokines and chemokines, impacts on lipid metabolism, and effects on the immune system. These changes collectively demonstrate the mechanisms through which intestinal flora imbalance exacerbates UC.

Disorder of the intestinal flora also plays a significant role in the pathogenesis of UC. Under normal circumstances, the intestinal flora and the host are in a symbiotic state, which is crucial for maintaining intestinal homeostasis. However, in UC patients, the structure of the intestinal flora undergoes significant changes, characterized by an increase in the number of pathogenic or opportunistic pathogenic bacteria such as adherent-invasive *Escherichia coli* (AIEC), while the number of beneficial bacteria such as *Bifidobacterium* and *Lactobacillus* decreases [10]. This imbalance of microbiota can trigger inflammation through multiple pathways. For instance, pathogenic bacteria can directly adhere to and invade intestinal epithelial cells, damaging the intestinal barrier. Their metabolic products, such as lipopolysaccharide (LPS), can activate pattern recognition receptors like toll-like receptors (TLRs), thereby initiating downstream inflammatory signaling pathways [11]. At the same time, dysbiosis of the microbiota can also affect the metabolic environment of the intestine, leading to a reduction in protective metabolic products such as short-chain fatty acids (SCFAs), and weakening the anti-inflammatory and repair capabilities of the intestine [12] (Figure 2).

Intestinal barrier injury is an important basis for the onset of UC. The physical barrier formed by intestinal epithelial cells, the mucus layer secreted by goblet cells, and the immune barrier composed of immune cells jointly maintain the integrity of the intestinal barrier. In UC patients, intestinal epithelial cells are damaged and die due to attacks from inflammatory factors, oxidative stress, and other factors. The expression of tight junction proteins such as occludin and claudin between cells decreases or becomes abnormal, leading to increased intestinal barrier permeability [13]. The number of goblet cells decreases and their function is impaired, resulting in insufficient mucus secretion and a thinner or broken mucus layer, which fails to effectively prevent direct contact between intestinal flora and intestinal epithelial cells. The damage to the intestinal barrier allows harmful substances such as intestinal flora and toxins to invade the submucosa of the intestine, further activating immune cells and triggering a more intense inflammatory response. Inflammation, in turn, aggravates the damage to the intestinal barrier, creating a vicious cycle [14].

## 3. The Mechanism of PANoptosis

PANoptosis is a novel type of inflammatory programmed cell death that was discovered in recent years. Unlike traditional forms of cell death such as apoptosis, pyroptosis, and necroptosis, it is characterized by the simultaneous activation of these death pathways and the release of a large amount of pro-inflammatory cytokines. It plays a significant role in the body’s immune defense and in the pathological processes of various diseases (Table 1). The core of its molecular mechanism is the formation of the PANoptosome complex [15]. This complex is a multi-protein complex that can integrate key molecules from various death signaling pathways, including inflammasomes (such as NLRP3 and NLRC4), caspase family members (such as caspase-1 and caspase-8), receptor-interacting protein kinase (RIPK) family members (such as RIPK1 and RIPK3), Z-DNA binding protein 1 (ZBP1, also known as DNA-dependent activator of interferon regulatory factors DAI), etc. When cells are stimulated by external factors, these molecules interact through specific domains to assemble this complex, thereby initiating a PANoptotic signaling cascade [16]. During signal transduction, the activation of inflammasomes is a crucial step. As intracellular pattern recognition receptor complexes, they can recognize pathogen-associated molecular patterns (PAMPs) and damage-associated molecular patterns (DAMPs). After activation, they recruit caspase-1 and cause its autocatalytic activation. Activated caspase-1 can cleave the precursors of interleukin-1β (pro-IL-1β) and interleukin-18 (pro-IL-18), generating the mature pro-inflammatory cytokines IL-1β and IL-18 and releasing them outside the cell. Activated caspase-1 can also interact with other molecules to activate apoptosis- and necroptosis-related signaling pathways [17]. Caspase-8 plays a crucial pivotal role, serving as an important executor of the apoptotic pathway by cleaving downstream substrates to trigger apoptosis. It also interacts with molecules such as RIPK1 and RIPK3 to regulate the occurrence of necroptosis, and it participates in the activation of inflammasomes to further promote the progression of PANoptosis. RIPK1 and RIPK3 mainly activate mixed lineage kinase domain-like protein (MLKL) by forming necrosomes, phosphorylating MLKL and transferring it to the cell membrane, causing membrane perforation, triggering necroptosis and releasing pro-inflammatory factors [18]. The triggers for the activation of PANoptosis are diverse and include pathogen infection (when bacteria or viruses invade, the PAMPs they carry are recognized by intracellular pattern recognition receptors, which trigger PANoptosis to eliminate the pathogens), cell damage (DAMPs released after damage, such as ATP, high mobility group box 1 protein (HMGB1), etc., activate PANoptosis signals to trigger tissue damage repair or regulate inflammatory responses), and cell stress (oxidative stress, DNA damage and other signals induce PANoptosis to eliminate damaged cells) [19]. Four classic PANoptosome complexes have been identified so far, namely ZBP1, AIM2, RIPK1, and NLRP12. Among them, the ZBP1-PANoptosome mainly consists of ZBP1, NLRP3, ASC, caspase-1/6/8, and RIPK1/3. ZBP1 can activate interferon (IFN) and nuclear factor κB (NF-κB) signals, and also regulate inflammation and cell death through the RIPK1–RIPK3–caspase-8 pathway. Moreover, after NLRP3 and caspase-8 are activated, ASC can be recruited to promote the self-activation of caspase-1 and the secretion of IL-1β [20]. The AIM2-PANoptosome contains AIM2, Pyrin, ZBP1, ASC, caspase-1/8, RIPK1/3, and FADD. AIM2, as a DNA sensor, recruits ASC and caspase-1 upon activation. Its deficiency reduces the expression of Pyrin and ZBP1. Pyrin deficiency partially reduces PANoptosis. FADD, as a caspase-8 adaptor, participates in caspase-8-dependent inflammatory responses and can also suppress inflammation by inhibiting ZBP1 and necroptosis expression, as well as caspase-8-GSDMD-dependent pyroptosis and MLKL-induced necroptosis in epithelial cells. Caspase-8 can also act independently of FADD in certain circumstances [21]. The RIPK1-PANoptosome forms during Yersinia infection and contains RIPK1, RIPK3, caspase-1/8, NLRP3, and ASC. RIPK1, as the main regulatory factor of TNFR1 signaling, can promote the transcription of inflammatory cytokines and regulate cell death. The NLRP12-PANoptosome contains NLRP12, ASC, caspase-8, and RIPK3. NLRP12 is crucial for the activation of inflammasomes and PANoptosomes induced by heme and PAMPs and it can also recognize Yersinia to resist bacterial infections. It plays an anti-inflammatory role in colonic inflammation and has an anti-tumor effect in colorectal cancer [22,23,24,25]. 

**Figure 3:**
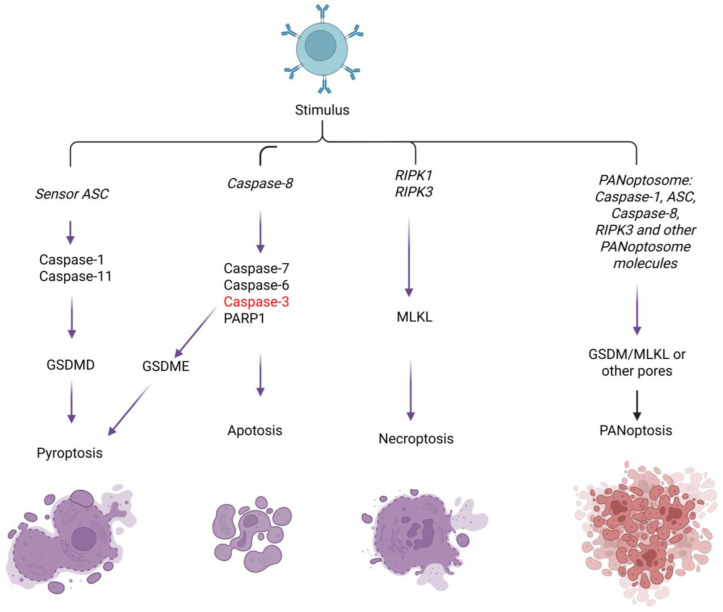
Extracellular stimuli activate distinct signaling pathways—including ASC/caspase-1/11, caspase-8, RIPK1/RIPK3, and the PANoptosome complex—to drive pyroptosis, apoptosis, necroptosis, and PANoptosis, highlighting the molecular coordination and crosstalk among programmed cell death pathways.

IFN regulatory factor 1 (IRF1) can positively regulate PANoptosis and the activation of multiple PANoptosomes. For instance, it mediates PANoptosis through TNF + IFN-γ stimulation, positively regulates the expression of ZBP1 and participates in the PANoptosis mediated by it, regulates the activation of AIM2 and cell death, acts as an upstream regulator of the RIPK1-PANoptosome in response to TAK1 inhibitor (TAKi) and lipopolysaccharide (LPS) stimulation, and can also mediate the formation of the NLRP12-PANoptosome [26]. During infection with influenza A virus (IAV), the DAI/sperm-associated antigen 9/c-Jun N-terminal kinase (DAI/SPAG9/JNK) signaling pathway can enhance the interaction between RIPK1, RIPK3 and DAI, promoting the formation of PANoptosomes [27,28]. However, the assembly mechanism of PANoptosomes is still rather complex. Currently, there are still unclarified sensor-specific complexes, and their specific roles and functions in PANoptosis need to be further explored (Table 2; Figure 3).

## 4. PANoptosis of Intestinal Epithelial Cells and Immune Cell Dysfunction in Ulcerative Colitis

The PANoptosis of intestinal epithelial cells is not merely a simple form of cell death, but rather a cascade of structural damage, signal release and immune activation, which drives the progression of UC [29]. Intestinal epithelial cells are stimulated by lipopolysaccharide (LPS), flagellin and other microbe-associated molecular patterns (MAMPs) through interactions with intestinal microbiota, which activate the NLRP3 inflammasome within the cells [30]. The activation of the NLRP3 inflammasome triggers caspase-1-mediated cleavage of the GSDMD (Gasdermin D) protein, causing pore effects. This process disrupts the membrane structure of intestinal epithelial cells, making the cell membrane highly permeable and ultimately leading to leakage of intracellular contents [31]. This not only directly leads to increased permeability of intestinal epithelial cells but also triggers a series of immune responses, promoting further inflammation. During this process, the physical barrier function of intestinal epithelial cells is impaired, resulting in the disruption of the integrity of the intestinal barrier. Besides the direct barrier defect caused by cell shedding, the degradation of tight junction proteins (such as ZO-1 and claudin-1) further aggravates the barrier dysfunction [32]. Studies have shown that the expression level of claudin-1 in the intestinal mucosa of UC patients is approximately 52% lower than that in healthy individuals, and this reduction is negatively correlated with the activation level of GSDMD [30]. This indicates that the GSDMD-mediated PANoptosis process is closely related to the degradation of tight junction proteins in intestinal epithelial cells and plays a significant role in the pathological process of UC. This cascade reaction not only exacerbates the intestinal inflammatory response through cell death and the loss of intestinal barrier function, but also forms a vicious cycle through the activation of the immune system, further promoting the chronicity and recurrence of UC [3]. Therefore, targeting GSDMD activation, restoring intestinal barrier function and regulating immune responses may become important strategies for treating UC. By interfering with this PANoptotic process and immune activation pathway, it is expected to alleviate the pathological symptoms of UC and provide new therapeutic ideas.

**Figure 4:**
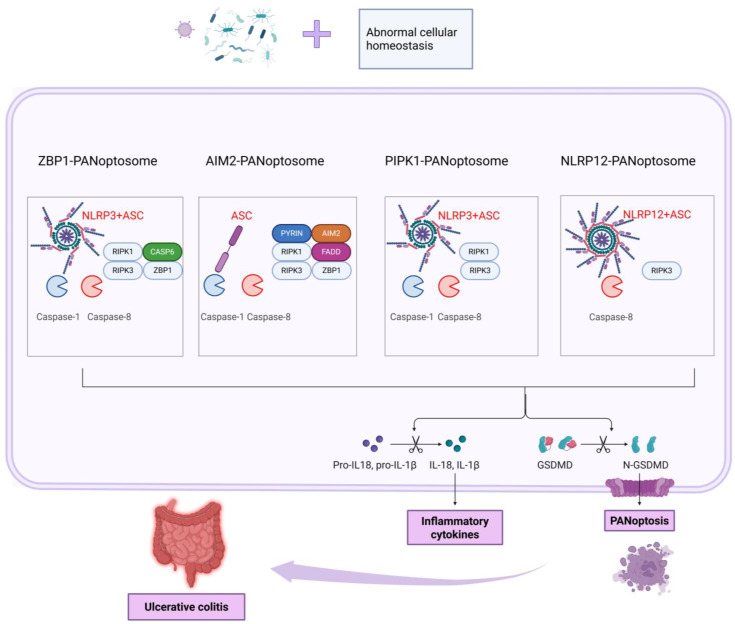
This figure illustrates the molecular mechanism through which ulcerative colitis develops. Under the combined effect of intestinal flora abnormalities and abnormal cellular homeostasis, four different PANoptosome complexes (ZBP1-PANoptosome, AIM2-PANoptosome, PIPK1-PANoptosome, and NLRP12-PANoptosome) activate molecules such as Caspase-1 and Caspase-8. This leads to the cleavage of Pro-IL18 and Pro-IL1β into inflammatory cytokines like IL-18 and IL-1β for release. Meanwhile, it drives the cleavage of GSDMD into N-GSDMD, triggering PANoptosis (inflammatory cell death). Ultimately, these processes result in the occurrence of ulcerative colitis.

During the process of PANoptosis, the release of DAMPs from the contents of intestinal epithelial cells further intensifies the inflammatory response and amplifies the immune activation signals [33]. After cell death, molecules such as ATP are released extracellularly as DAMPs. These DAMPs can activate the P2X7 receptor, initiating the assembly of the NLRP3 inflammasome in macrophages and dendritic cells, thereby promoting the release of more pro-inflammatory factors. This effect of ATP not only enhances the local immune response, but also contributes to the maintenance of chronic inflammation [34]. Meanwhile, another important DAMP molecule, high mobility group box 1 protein (HMGB1), is also released in PANoptotic cells. HMGB1 activates the NF-κB signaling pathway by binding to the TLR4 receptor, prompting immune cells such as macrophages and dendritic cells in the intestine to secrete large amounts of pro-inflammatory factors such as IL-6 and TNF-α [35]. These pro-inflammatory factors not only recruit circulating immune cells to infiltrate the intestinal mucosa, further intensifying the immune response in the intestine, but also, through self-amplification, act on intestinal epithelial cells, leading to the sustained activation of inflammation. While these factors trigger the infiltration of immune cells, they also form a positive feedback loop of “PANoptotic and inflammation” by acting on intestinal epithelial cells and the intestinal immune system [36]. Through this mechanism, the immune response triggered by PANoptotic not only aggravates the damage to the intestinal barrier but also promotes the persistence of chronic intestinal inflammation and may exacerbate the pathological progression of UC. Therefore, the inhibition of DAMPs, the regulation of the NLRP3 inflammasome, and targeting the NF-κB pathway may provide new therapeutic targets for alleviating UC symptoms and repairing the intestinal barrier (Table 3). PANoptosis likely functions as a pathogenic amplifier within a feed-forward loop, rather than an exclusive primary driver of UC. This bidirectional relationship suggests that inhibiting PANoptosis may interrupt the inflammatory cycle but may not eliminate upstream disease triggers [37] (Figure 4).

It should be noted that several mechanistic uncertainties remain unresolved in the current literature. For instance, the relative contribution of different PANoptosome sensors (ZBP1, AIM2, RIPK1, and NLRP12) may vary across cell types and stimuli, and direct evidence distinguishing PANoptosis from concurrent but independent activation of pyroptosis, apoptosis, and necroptosis is still lacking. These uncertainties warrant further investigation.

## 5. Molecular Mechanisms of Immune Cell PANoptosis and Pro-Inflammatory Dominance

### 5.1. The Phenotypic Imbalance of Macrophages Is Exacerbated

The polarization of M1 macrophages is precisely regulated by PANoptotic signals. When macrophages phagocytose apoptotic bodies released by PANoptotic intestinal epithelial cells, the NLRP3/caspase-1 pathway within them is activated, promoting the expression of M1 phenotype markers (such as CD86 and iNOS) by up-regulating the IRF5 transcription factor [38]. Meanwhile, the differentiation of M2-type macrophages is inhibited in a dual manner: on the one hand, the release of IL-1β through PANoptosis can block the IL-4/STAT6-mediated M2 polarization pathway; on the other hand, the secretion of TGF-β1 is reduced by 38%, resulting in a significant decline in its ability to repair the intestinal mucosa [39]. One study reported that this ratio increased by 4.2-fold in macrophages from UC patients compared to healthy controls, contributing to a pro-inflammatory microenvironment [40].

### 5.2. The Imbalance and Functional Abnormalities of T Cell Subsets

The abnormal activation of Th17 cells depends on a cytokine network related to PANoptosis. IL-1β and IL-23 released by PANoptotic intestinal epithelial cells can synergistically activate the RORγt transcription factor within Th17 cells, increasing their proliferation rate by 2.8 times and promoting the secretion of cytokines such as IL-17A and IL-22 in large quantities [41]. Among them, IL-17A specifically recruits neutrophils by inducing the expression of chemokines such as CXCL1 and CXCL2 in intestinal epithelial cells, and simultaneously stimulates epithelial cells to produce matrix metalloproteinases (MMP-3 and MMP-9), leading to the degradation of the intestinal mucosal basement membrane [42]. In contrast, the immunosuppressive function of Treg cells is impaired by the PANoptotic signal; IL-6 released by PANoptosis reduces the expression of CTLA-4 and Foxp3 on Treg cells through activating the STAT3 pathway, thereby decreasing their ability to suppress Th17 cells by approximately 60%, as validated in specific experimental conditions such as mouse colitis models and in vitro co-culture systems [43,44]. In addition, under LPS stimulation, Treg cells themselves can also undergo PANoptosis. The cleavage of GSDMD in Treg cells increases, leading to a reduction in their number and the release of pro-inflammatory factors, further weakening the immune regulatory network [33,45].

### 5.3. The “Over-Defense” of Neutrophils and Tissue Damage

Neutrophils exhibit a paradoxical state of “hyperfunction and self-damage” under PANoptosis-driven conditions. On the one hand, IL-1β associated with PANoptosis can enhance the expression of Fc receptors and complement receptors on the surface of neutrophils, increasing their phagocytic capacity by 1.7 times [46]. On the other hand, the accumulated evidence indicates that ROS levels are significantly elevated in the colonic biopsy tissues of UC patients compared with healthy individuals. This excessive oxidative stress is consistent with the pathological features of ulcerative colitis, though the specific degree of elevation varies with disease activity and sampling location [47]. These ROS not only damage intestinal epithelial cells through oxidative stress, but also activate the NETosis program of neutrophils, releasing neutrophil extracellular traps (NETs) that contain DNA, histones and elastase [48]. The dual role of NETs is particularly prominent in UC. While they can capture pathogens, the excessive release of NETs can directly degrade the mucin layer of the intestinal mucosa (such as MUC2), exposing the epithelial cells to attack from the microbiota [49]. Research findings indicate that the levels of NETs markers (such as citrullinated histone H3) in the intestinal mucosa of UC patients are positively correlated with the mucosal injury score (r = 0.63, *p* < 0.001), and their elastase can degrade the tight junction protein occludin, forming a vicious cycle of “barrier disruption, neutrophil infiltration, and further disruption” [49,50].

### 5.4. The Cascade Amplification Effect of the Cytokine Network

The above-mentioned mechanism eventually forms a “pro-inflammatory dominance” through a cascade reaction of cytokines. IL-17 secreted by Th17 cells and TNF-α released by macrophages act in synergy, increasing the expression of IL-8 in intestinal epithelial cells by five times and continuously recruiting neutrophils [51,52]. However, the secretion of IL-10 by M2 macrophages and Treg cells is insufficient (only 40% of the healthy level), which is unable to effectively inhibit the activation of the NF-κB and MAPK pathways [53]. This “hyperactivity of pro-inflammatory factors and depletion of anti-inflammatory factors” imbalance leads to the transformation of acute inflammatory damage to the intestinal mucosa into a chronic and persistent state, which becomes the core pathological basis for the difficulty in curing UC.

## 6. Therapies Targeting PANoptosis

### 6.1. Targeting PANoptosis of Intestinal Epithelial Cells Alleviates UC

Targeting PANoptosis in intestinal epithelial cells is one of the important strategies for alleviating UC. Small molecule inhibitors have shown certain potential in this regard. For instance, RIPK1 inhibitors (such as Nec-1s) can specifically inhibit the activity of RIPK1, block the transmission of PANoptosis signaling pathways, reduce PANoptosis in intestinal epithelial cells, and thereby protect the integrity of the intestinal barrier [54]. Studies have shown that in UC animal models, the use of Nec-1s can significantly alleviate the inflammatory damage to the intestinal mucosa and reduce the levels of pro-inflammatory factors.

Natural products have also been found to be able to inhibit PANoptosis of intestinal epithelial cells. Curcumin, an active component extracted from turmeric, possesses multiple biological activities such as anti-inflammatory and antioxidant effects [55]. It can reduce the PANoptosis of intestinal epithelial cells by inhibiting the activation of inflammasomes and regulating related signaling pathways, effectively alleviating the symptoms and inflammatory degree of UC in experimental models [56]. In addition, other natural products such as resveratrol have also been confirmed to inhibit the PANoptosis of intestinal epithelial cells through a similar mechanism, and are expected to become potential drugs for the treatment of UC [57].

### 6.2. Targeting Immune Cell Dysfunction Alleviates UC

Therapeutic measures targeting the immune cell dysfunction caused by PANoptosis could alleviate UC. To target the macrophage dysfunction, TLR4 inhibitors can be used. By inhibiting the TLR4 signaling pathway, the polarization of M1-type macrophages and the secretion of pro-inflammatory factors can be reduced while promoting the differentiation of M2-type macrophages and enhancing the anti-inflammatory effect [58]. To target the abnormal T-cell function, IL-17 antagonists can be applied. These antagonists can specifically bind to IL-17, block its biological activity, and inhibit the pro-inflammatory response mediated by Th17 cells [59]. Clinical research has shown that IL-17 antagonists can significantly increase the clinical remission rate and improve symptoms in patients with moderate to severe UC. Additionally, by regulating the function of Treg cells, such as promoting their proliferation and activation with cytokines like IL-2 to enhance their immunosuppressive effects, it could restore immune balance and alleviate the inflammation that occurs in UC [60].

### 6.3. Multi-Target Inhibition Strategies for PANoptosis and Inflammatory Cell Death

In mammals, the caspase family consists of 14 members, which can be classified into apoptotic subtypes (such as caspase-3 and 6–10) and inflammatory subtypes (such as caspase-1, 4, 5 and 11) [61]. They not only mediate apoptosis, but also participate in various cell death pathways induced by damage-associated molecular patterns (DAMPs) or pathogen-associated molecular patterns (PAMPs) and human diseases. For instance, caspase-6 can bind to RIPK3, enhancing the interaction between ZBP1 and RIPK3, thereby activating the PANoptotic pathway [62]. Drugs such as the caspase-1 inhibitor VX-765 [63], CZL80 [64,65] and tetracycline [66] can alleviate inflammatory responses by inhibiting the production of caspase-1-dependent cytokines. Specific inhibitors of NLRP3, the most representative inflammasome sensor, include MCC950 [67,68], OLT1177 (dapansutrile) [69], glibenclamide [70,71] and its analogues JC171 [72], CY-09 [73], BOT-4-one [74], Fc11a-2 [75], etc., which inhibit the activation of NLRP3 through different mechanisms, thereby blocking the release of IL-1β and IL-18. Some of them have already entered the clinical trial stage. Meanwhile, RIPK1, as a key molecule regulating cell survival and death, has various types of inhibitors, including type I (such as tozasertib [76]), type II and type III inhibitors (such as GSK963 [77], GSK772 [78], DNL104 [79], GFH312 [80], etc.). Many of these compounds have shown good efficacy in inflammatory and degenerative disease models, and some have entered clinical trials. Inhibitors of RIPK3, a key kinase in necroptosis, include AZD5423 [81], CPD42 [82], and HS-1371 [83], which exert protective effects by blocking the RIPK1–RIPK3–MLKL pathway. In addition, research on other pan-apoptosis-related proteins such as ZBP [84,85], AIM2 [86], ASC [87], and the pore-forming molecules GSDMD (whose inhibitors include disulfiram [88], LDC7559 [89], etc.) and MLKL (such as NSA [90]) has also made progress. These molecules regulate multiple cell death pathways including pyroptosis, apoptosis, and necroptosis by interfering with the assembly of the PANoptosome complex or downstream effector mechanisms. Overall, the development of inhibitors targeting cell death pathways should take into account the interactions between multiple pathways. Future research should focus on evaluating their comprehensive effects in a PANoptotic context to achieve more effective treatment of inflammation and diseases.

### 6.4. Innovative Therapy for UC Targeting PANoptosis

Given that the activation of PANoptosis in UC involves complex signaling pathways, which limits the efficacy of single-target therapy, an intelligent response-type hydrogel delivery system loaded with multiple PANoptosis inhibitors has emerged, providing an innovative direction for precise and synergistic treatment of UC [91]. The core of this system is an oral- or colon-targeted hydrogel composed of biocompatible materials such as chitosan and sodium alginate, which has the functions of lesion-localized enrichment, environment-responsive release (in response to specific pH or enzyme environments in the intestine), and serving as a synergistic drug delivery platform [92,93,94]. The “inhibitor cocktail” it carries can systematically intervene in the PANoptotic cascade: upstream, the cocktail inhibits the NLRP3 inflammasome through the action of MCC950 or CY-09, inhibits RIPK1 through GSK772 or Nec-1s, and interferes with the ZBP1 pathway to block the assembly of the PANoptosome complex [95]. Downstream, the VX-765 in the cocktail inhibits caspase-1, while disulfiram and NSA suppress the pore-forming activities of GSDMD and MLKL, respectively, to curb the pyroptosis and necroptosis of cells, protecting the integrity of the intestinal barrier and reducing the release of damage-associated molecular patterns (DAMPs) [19,96]. This multi-drug combination strategy achieves synergistic effects through “vertical blocking” (inhibition at different levels of the same pathway) and “horizontal coverage” (simultaneously targeting different branches of the cell death pathways), fundamentally breaking the vicious cycle of “cell death, inflammation and more cell death”. It represents a cutting-edge therapy that efficiently inhibits PANoptosis, alleviates intestinal inflammation, and promotes mucosal healing through a multi-target, localized approach. Although it still faces challenges in the co-encapsulation and precise controlled release of drugs, it undoubtedly opens up a promising new path for conquering refractory UC. It is important to emphasize that no published studies have evaluated the exact combination of inhibitors proposed here in UC models. The selection of inhibitors (MCC950 for NLRP3, GSK772 for RIPK1, VX-765 for caspase-1, disulfiram for GSDMD, and NSA for MLKL) is based on their complementary mechanisms of action targeting distinct nodes within the PANoptosis network, and the combination strategy requires systematic validation in preclinical settings. This multi-drug combination strategy is hypothesized to achieve synergistic effects through vertical blocking (inhibition at different levels of the same pathway) and horizontal coverage (simultaneously targeting different branches of the cell death pathways), potentially breaking the vicious cycle of cell death, inflammation and more cell death. This hypothesis, while mechanistically rational, awaits experimental validation in UC models.

While this multi-target hydrogel strategy offers theoretical advantages, it also raises important safety considerations that must be addressed before clinical translation. (1) Oncogenic risk: UC patients already have an increased risk of colorectal cancer, and sustained inhibition of multiple cell death pathways, particularly apoptosis, could theoretically impair the elimination of transformed cells. Long-term safety studies in carcinogen-induced or genetic colorectal cancer models will be essential to exclude tumor-promoting effects. (2) Infection susceptibility: Many cell death pathways play critical roles in host defenses against enteric pathogens. Broad inhibition of PANoptosis might increase susceptibility to bacterial, viral, or fungal infections, a risk that must be evaluated in infectious challenge models. (3) Impaired tissue homeostasis: Physiologic cell death is essential for intestinal epithelial renewal and immune homeostasis. The consequences of sustained regional inhibition remain poorly understood and require careful evaluation of intestinal architecture and barrier function over extended treatment periods. (4) Off-target effects: The selected inhibitors may have activity beyond their intended targets. For example, disulfiram has multiple known off-target effects. Local delivery via hydrogel may mitigate systemic toxicity but may not eliminate local off-target risks. We emphasize that the proposed multi-inhibitor hydrogel remains a hypothetical concept requiring extensive preclinical safety evaluation before any clinical consideration (Table 4).

Although numerous inhibitors have shown efficacy in preclinical models, conflicting results have been reported depending on experimental conditions, and clinical translation has faced challenges such as a lack of biomarkers for patient selection. It should be noted that this multi-target hydrogel strategy currently remains a hypothetical concept. Several translational limitations must be addressed before clinical consideration, including the technical challenges of co-encapsulating multiple inhibitors with different physicochemical properties, achieving precise and sequential drug release, and the lack of safety data regarding long-term use in inflamed colonic tissue.

## 7. Conclusions

This review systematically described the key role of PANoptosis in the occurrence and development of UC: the complex pathological environment of UC provides multiple triggers for the activation of PANoptosis, and once initiated, the PANoptosome complex assembled by key molecules such as ZBP1, NLRP3, and RIPK1 will synergistically activate the caspase family and RIPKs, leading to the concurrent occurrence of pyroptosis, apoptosis, and necroptosis. This not only directly causes the death of intestinal epithelial cells and the breakdown of the physical barrier, but also further aggravates the dysfunction of immune cells by releasing a large amount of pro-inflammatory cytokines and DAMPs, forming a “cell death, inflammation” vicious cycle that drives the chronicity and refractoriness of UC. The redundancy of this signaling network makes the efficacy of traditional single-target strategies limited, highlighting the urgency and importance of developing multi-target combined intervention strategies. Looking to the future, the development of this field is full of opportunities and challenges. To discover new inhibitors and optimize combined strategies, high-throughput screening and artificial intelligence-assisted design should be utilized to discover specific inhibitors for key sensors such as ZBP1 and AIM2, and the best combination drug regimens should be scientifically determined. To improve the precision and functionalization of intelligent delivery systems, more intelligent materials responsive to the characteristics of the UC microenvironment (such as reactive oxygen species levels and enzyme profiles) should be developed, and a multifunctional platform integrating “inhibiting death, reducing inflammation, repairing the barrier and regulating the microecology” should be constructed. Regarding clinical translation, the safety and efficacy of multi-target hydrogel systems should be systematically verified in advanced animal models, and early clinical trials for refractory patients should be carefully designed. To understand the basic mechanisms of PANoptosis, the precise assembly mechanism of PANoptosomes and their dynamic regulatory network in different cell types and clinical subtypes must be extensively studied. In summary, by combining multi-target inhibition strategies with advanced delivery technologies and by deepening our understanding of the basic mechanisms of PANoptosis, we can expect the development of breakthrough therapies that can fundamentally break the pathological vicious cycle of UC. A critical limitation in the current literature is the lack of direct clinical evidence demonstrating PANoptosome complex assembly in human UC biopsies. Most mechanistic insights are derived from in vitro cell systems and animal models. Future translational studies should prioritize: (1) the development of validated in situ detection methods for PANoptosome components, and (2) prospective cohort studies correlating the expression of PANoptosis-related molecules with clinical disease activity indices, endoscopic severity, and treatment response.

## Figures and Tables

**Table 1:** Core molecular mechanisms of PANoptosomes.

Mechanism	Key Molecule/Pathway	Core Functions and Roles
Core platform	PANoptosome complex	The multi-protein complex integrating key proteins of the apoptosis, pyroptosis and necroptosis signaling pathways is the core signal hub that initiates pan-apoptosis.
Key sensors and effector molecules	Inflammasomes (such as NLRP3)	As an intracellular pattern recognition receptor complex, it assembles upon recognition of PAMPs/DAMPs and recruits and activates caspase-1.
	Caspase-1	Cuts GSDMD to induce pyroptosis of cells; processes pro-IL-1β and pro-IL-18 into mature pro-inflammatory factors and releases them.
	Caspase-8	Key hub molecule: As an executor of apoptosis, it cleaves downstream substrates; interacts with RIPK1/RIPK3 to regulate necroptosis; participates in the activation of inflammasomes.
	The RIPK1/RIPK3/MLKL pathway	RIPK1 and RIPK3 form “necrosomes”, which phosphorylate and activate MLKL, leading to its oligomerization and pore formation in the cell membrane, thereby executing necroptosis.
Activate the trigger	Pathogen infection	PAMPs (such as nucleic acids and LPS) provided by viruses, bacteria, etc., are recognized by intracellular sensors.
	Cellular damage and stress	Signals such as DAMPs (e.g., ATP, HMGB1) are released by damaged cells due to oxidative stress and DNA damage.
Upstream regulatory factors	IRF1	It positively regulates the assembly and activation of multiple PANoptosomes, such as up-regulating the expression of ZBP1 in response to TNF + IFN-γ, TAK1 inhibitors and other stimuli.
	DAI/SPAG9/JNK pathway	During infection with influenza A virus, it enhances the interaction between RIPK1, RIPK3 and the sensor DAI (ZBP1) to promote the formation of PANoptosomes.

**Table 2:** Types and compositions of classic PANoptosome complexes.

PANoptosome Type	The Main Constituent Molecules	Specific Functions and Background of Action
ZBP1-PANoptosome	ZBP1 (DAI), NLRP3, ASC, caspase-1/6/8, RIPK1/3	ZBP1 senses viral nucleic acids, activates IFN and NF-κB signaling, and regulates death and inflammation through the RIPK1–RIPK3–caspase-8 axis.
AIM2-PANoptosome	AIM2, Pyrin, ZBP1, ASC, caspase-1/8, RIPK1/3, FADD	AIM2, as a cytoplasmic DNA sensor, activates the inflammasome; its deficiency affects the expression of Pyrin and ZBP1; FADD is involved in regulating the activity of caspase-8.
RIPK1-PANoptosome	RIPK1, RIPK3, caspase-1/8, NLRP3, ASC	Assembles in response to TNFR1 signaling or Yersinia infection; regulates inflammatory cell death and cytokine production.
NLRP12-PANoptosome	NLRP12, ASC, caspase-8, RIPK3	NLRP12 senses heme and PAMPs and other signals, and plays a regulatory role in resisting Yersinia and in colon-related diseases.

**Table 3:** Pathological mechanism of PANoptosomes in intestinal epithelial cells in ulcerative colitis.

Pathological Stage	Key Events and Molecules	Consequences and Significance
Initiation and Direct Damage	Stimulation: Intestinal microbiota MAMPs (such as LPS) activate the NLRP3 inflammasome in intestinal epithelial cells. Execution: Caspase-1 cleaves GSDMD, leading to pyroptosis and leakage of intracellular contents. Structural damage: Tight junction proteins (such as claudin-1) are degraded, resulting in the loss of the physical barrier.	The integrity of the intestinal epithelium is disrupted, leading to the formation of initial lesions and barrier defects.
Amplification of Inflammatory Signals	DAMP release: Dying cells release ATP, HMGB1, etc. Immune cell activation: ATP activates the NLRP3 inflammasome in macrophages/dendritic cells through the P2X7 receptor, releasing IL-1β, etc.; HMGB1 activates the NF-κB pathway in macrophages/dendritic cells through TLR4, secreting large amounts of TNF-α, IL-6, etc.	The local inflammatory response is rapidly amplified, recruiting more immune cells to infiltrate and forming a strong inflammatory microenvironment.
Vicious Circle is Formed	Positive feedback loop: Pro-inflammatory factors released by immune cells (such as TNF-α and IL-6) can act on intestinal epithelial cells, further promoting their death and the release of DAMPs.	Establishing a self-perpetuating cycle of “pan-apoptosis → barrier damage → DAMP release → immune activation → inflammation aggravation → more pan-apoptosis” promotes the chronicity and recurrence of UC.

**Table 4:** Therapeutic PANoptosis targets and their inhibitors for UC treatment.

Target Molecule	Inhibitor/Drug	Evidence Stage
NLRP3	MCC950, CY-09, OLT1177, glibenclamide	Preclinical/Phase II
RIPK1	GSK772, Nec-1s, DNL104, GFH312	Preclinical/Phase I
Caspase-1	VX-765, CZL80	Preclinical
GSDMD	Disulfiram, LDC7559	Preclinical
MLKL	NSA	Preclinical
RIPK3	AZD5423, CPD42, HS-1371	Preclinical
ZBP1	Under development	Preclinical
AIM2	Under development	Preclinical
ASC	Under development	Preclinical

## Data Availability

No new data were created or analyzed in this study. Data sharing is not applicable to this article.
